# ‘I think positivity breeds positivity’: a qualitative exploration of the role of family members in supporting those with chronic musculoskeletal pain to stay at work

**DOI:** 10.1186/s12875-015-0302-1

**Published:** 2015-07-22

**Authors:** Serena McCluskey, Haitze de Vries, Michiel Reneman, Joanna Brooks, Sandra Brouwer

**Affiliations:** Centre for Applied Psychological and Health Research, University of Huddersfield, Queensgate, Huddersfield HD1 3DH UK; Department of Health Sciences, Community and Occupational Medicine, University Medical Center Groningen, University of Groningen, Groningen, The Netherlands; Department of Rehabilitation Medicine, Center for Rehabilitation, University Medical Center Groningen, University of Groningen, Groningen, The Netherlands

**Keywords:** Significant others, Family, Work, Chronic pain, Musculoskeletal disorders, Psychosocial

## Abstract

**Background:**

It is proposed that family members are important sources of support in helping those with chronic musculoskeletal pain to remain at work, but the phenomenon remains largely unexplored. The aim of this study was to examine the extent and nature of support provided by family members in this respect.

**Methods:**

Qualitative data were collected from workers and their ‘significant others’ (spouses/partners/close family members) in two un-related studies focused on working with pain; one conducted in the United Kingdom (n = 10 dyads) and one in the Netherlands (n = 21 dyads). Thematic analysis techniques were applied to both sets of data independently, and findings were then assimilated to establish common themes.

**Results:**

Findings were broadly similar in both studies. Workers acknowledged significant other support in helping them to manage their pain and remain at work, and their descriptions of the type of support provided and required were echoed by their significant others. Three common themes were identified - ‘connectivity’, ‘activity’ and ‘positivity’. Worker and significant other responses were largely congruent, but significant others provided more in-depth information on the nature of their support, their concerns and the impact on their relationship.

**Conclusions:**

This research presents novel insights about the specific contribution made by significant others in helping their relatives with chronic musculoskeletal pain to stay at work. These findings add to the under-represented ‘social’ dimension of the biopsychosocial model currently applied to our understanding and treatment of pain, and point to harnessing support from significant others as a potentially effective management strategy.

## Background

Sickness absence and work disability due to chronic musculoskeletal pain (CMP) represents a major public health concern, and current healthcare practice is focused on supporting individuals with CMP to remain at work. CMP can be influenced by environmental factors, with an important source being the interaction between the pain sufferer and their ‘significant other’ (spouse/partner/close family member) [1, 2]. Several studies have proposed that significant others can reinforce an individual’s unhelpful pain cognitions, such as fear of movement, catastrophizing thoughts about pain and recovery, mistaken beliefs about the nature of pain, pessimistic beliefs regarding the outcome of treatment, and the unlikelihood of returning to work [3–6]. It has also been shown that potentially detrimental pain behaviors, such as over-use of pain medication, disturbed gait or limping, unduly resting/lying down, seeking compensation, or absence from work, can persist due to the overly-solicitous and/or negative responses of significant others [7–9].

However, the majority of this research is largely conducted with those who report a high degree of disability, probably resulting in an unrepresentative focus on the negative influences of significant others. Recent research conducted with individuals who have managed to remain at work with CMP has indicated that significant others may act as a positive reinforcement and be a valuable source of support [10–12]. Further understanding of the supportive role of significant others in this context may usefully inform more effective means of assisting those with CMP to deal with the behavioural and affective components of their condition, aligning with the evidence which suggests that coping strategies are most effective when employed in collaboration with, and capitalizing on, an individual’s sources of ongoing social support [13]. Despite the proposed importance of significant others in this process, some gaps in the evidence remain. Firstly, the majority of data investigating the influence of significant others on CMP is either collected from individuals with pain reporting *their own* perceptions of their significant other’s beliefs and behaviours, or significant other behaviours are observed and reported by a researcher or clinician – such data is rarely collected from significant others themselves.

Therefore, in an attempt address these gaps in the evidence and to elaborate further on the under-explored supportive role of significant others in helping those with CMP to remain at work, qualitative data collected from both workers with CMP and their significant others in two un-related studies focused on working with CMP conducted in the United Kingdom (UK) and the Netherlands were assimilated. The aim of this study was to examine the extent and nature of support provided by significant others.

## Methods

### Design

This study was a secondary analysis of qualitative data collected in two un-related studies conducted in the UK and the Netherlands, both of which were focused on understanding the lived experience of working with CMP and how wider social circumstances could influence work participation (hence the inclusion of ‘significant others’). Secondary analysis of qualitative data is recognised as a valuable way of gaining a new perspective on novel research topics, allowing researchers to perform additional analysis on concepts emerging from research and address these in more detail [14, 15].

In both studies (for comparison purposes), participants were included who had become work-disabled, but for this paper only data from those who had managed to stay at work were selected. Full details for both studies and their findings are reported elsewhere [16, 17] - the qualitative data presented in this paper come from a sub-section of open-ended questions exploring how significant others support workers with CMP to stay at work. Ethical approval was granted by National Health Service Research Ethics (reference number 11/H1302/6) in the UK, and by The Medical Ethical Committee of the University Medical Center Groningen (registration number NL23870.042.08) in the Netherlands. Written informed consent was obtained from all study participants.

### Setting and participants

#### The UK

Workers reporting persistent non-specific low back pain attending a hospital pain management clinic were recruited by their consultant, along with their significant others (n = 10 dyads).

#### The Netherlands

A sample of workers reporting persistent musculoskeletal pain were recruited via announcements in newspapers and websites of national patient associations of back pain, whiplash and fibromyalgia, along with their spouses/partners (n = 21 dyads). Data from all participants (not just those with back pain) were selected for assimilation.

### Data collection

#### The UK

Semi-structured interviews were conducted separately with workers and their significant others and open-ended questions were asked about the nature of support provided by significant others. Workers were asked “Could you tell me about anything your significant other does to help you manage your condition and stay at work?”, and “Are there any ways in which your significant other is unhelpful in this respect?” Significant others were also asked these questions, but wording was changed to relate to their perceptions and experience, e.g. “Could you tell me about what you do to help XX manage their condition and stay at work?”

#### The Netherlands

Open-ended questions were administered to a sample of worker and significant other participants in order to further investigate the support of significant others. The workers were asked: “How did others contribute to your ability to manage and stay at work with pain?” and their significant others were asked: “What was your role (if any) in helping your spouse/partner to manage and remain at work?” and “What would you advise other spouses/partners of workers with CMP to help them manage and stay at work?”

### Data analysis

Responses to the open-ended questions above were transcribed and data were analysed using thematic analysis techniques. Thematic analysis is used extensively as both an integral part of more in-depth qualitative methodologies and a method in its own right, and is recognised as a particularly appropriate method for exploratory qualitative work [18]. A set of procedural steps were undertaken by a member of each study team in accordance with published recommendations for conducting thematic analysis [19], and these were as follows: (1) a thorough familiarization of the interview data through reading and re-reading the transcripts (SM & HdV); (2) preliminary thematic coding of the data to tentatively define themes, whilst additionally recording any new themes which were identified as relevant (SM & HdV); and (3) organization of identified themes into meaningful clusters (SM). These data were then assimilated, checking that the pre-defined themes were valid for each set of data from the two separate studies, and reaching consensus on final themes (SM, JB & HdV). The final themes were checked and validated by an additional member of one of the study teams (MR), and verbatim extracts were selected to illustrate each theme.

## Results

### Participant characteristics

Participant characteristics were broadly similar in both UK and Dutch studies. All participants in the UK study had pain of the lower back, and this was the most reported pain condition in the Dutch study (50 %). All of the UK participants reported having had experienced their pain for at least 3 years, with the majority of Dutch participants reporting having had experienced their pain for at least 2 years (92 %). None of the UK participants reported work as the cause of their back pain, and neither did the majority of participants in the Dutch study (75 %). The mean age of study participants in the UK was 49.2 years for workers, and 36.6 years for significant others, and 49.0 and 50.2 respectively in the Dutch study. In the UK study, both worker and significant other participants were employed in professional occupations. In the Dutch study, 86 % of workers were employed in professional occupations, and 80 % of their partners were employed (of whom 47 % full-time, but occupation details were not collected), 11 % did voluntary work, and 9 % were retired. In both studies, the majority of worker participants were female, and the majority of significant other participants were male.

### Interview data

None of the workers in either study indicated that their significant others were unhelpful in any way, and all acknowledged their support in helping them to manage their pain and stay at work. All participants referred to help with everyday activities, but the responses revealed that emotional support, encouragement, and participating in joint activities were seen as crucial by both workers and significant others. Thus, three main themes were established: ‘connectivity’; ‘activity’ and ‘positivity’. These are further illustrated below.

#### 1. Connectivity

Workers felt that having understanding from someone at home was important, e.g.

*“Having someone who is understanding is something that you need, someone to take you seriously whatsoever, that is genuinely interested in you, and … well, empathize with you”; [NL, 36 years, male]*

*“It’s a big help having her there”; [UK, 45 years, male]*

*“She’s very sympathetic”. [UK, 50 years, male]*

Significant others elaborated on this aspect and suggested that maintaining communication about the pain, and that listening to the worker talking about the pain were seen as a way to help them cope, e.g.

*“Make sure that the complaints remain open to discussion”; [NL, 40 years, male]*

*“It is important to let them determine when to talk about the pain”; [NL, 54 years, male]*

*“Take the pain seriously, be patient, and avoid patronizing”; [NL, 47 years, male]*

*“You just listen really and just kind of sympathise, or sometimes try and change the subject and get her to think of some other things*”. *[UK, 39 years, male]*

Some significant others described how difficult this could be sometimes, but that certain allowances needed to be made, e.g.

*“Try to show understanding as much as possible…they might get grumpy because they are so tired from working and being in pain, but you have to be understanding”. [UK, 42 years, female]*

Within this theme, significant others revealed their anxiety about workers often not wanting to discuss their pain, e.g.

*“I am hardly involved, he discusses the pain rarely”; [NL, 59 years, female]*

*“My husband wants to do everything himself and does not usually talk about his pain. He is annoyed if you ask where it hurts and despite the pain he would prefer to do everything”; [NL, 57 years, female]*

*“We don’t really talk about it…whether it’s denial I don’t know”*; *[UK, 43 years, female]*

*“She doesn’t like letting people know, she covers it up”*; *[UK, 39 years, male]*

*“I think he worries about these things but he doesn’t say because he knows that I’ll worry*”; *[UK, 45 years, female]*

*“I’m worried….he prefers to ignore the pain and does not to ask for help”. [NL, 56 years, female]*

#### 2. Activity

The majority of workers reported that encouragement from significant others to keep active was important, e.g.

*“You need to have someone who pushes you to do something, it doesn’t matter what, whether it is to get me to physiotherapy, but someone who says ‘get off the bed and do something’”; [NL, 52 years, female]*

“*We walk together every morning at 5.45 am and that helps me more than anything”. [UK, 45 years, male]*

All significant others considered that to be able to remain at work with CMP, it was important to encourage workers to keep active, e.g.

*“Ensure that they remain active despite the pain”; [NL, 43 years, female]*

*“I tell him to continue with his activities and do not give in to the pain quickly ”*; *[NL, 51 years, male]*

*“Try to keep doing the things that are important and use your energy for that”*; *[NL, 43 years, male]*

*“Just continue, the pain is there, whether you work or not”; [NL, 58 years, male]*

*“If you’re at work, then you have no time to brood”; [UK, 42 years, female]*

*“Encourage them to exercise and carry on as normal is probably the absolute best”; [NL, 67 years, male]*

*“Find distraction in common interests like walking or cycling”. [UK, 25 years, female]*

Like workers, significant others also reported doing joint activities as a way of supporting the worker in their management of pain, but they went further to describe how this had also been positive for them and their relationship, e.g.

*“We both went every week to the local swimming pool for ages and learned to swim properly – that helped him and me because I probably wouldn’t have gone”; [UK, 43 years, female]*

*“I went walking with him at 5.45 am, it doesn’t take much and we both feel much better”; [UK, 42 years, female]*

*“We go out for lunch or we go for a walk and spending that time together has had a positive impact on our relationship”.[UK, 43 years, female]*

#### 3. Positivity

Most workers described the need for significant others to be a positive influence, e.g.

*“My husband always sees the positive side of anything, and I think I have taken that from him”; [UK, 46 years, female]*

*“You need a positive influence at home”; [NL, 36 years, male]*

*“And my husband then said to me, if you want it [to continue working] then you should go for it”. [NL, 54 years, female]*

In alignment with this, significant others also felt it was important to present a positive outlook and encourage positivity in workers, e.g.

*“Don’t be a whiner”; [NL, 36 years, male]*

*“Try to enjoy the things that you can and emphasize these. Go out to do fun things, to keep you socially involved”; [NL, 42 years, female]*

*“I always say, there are worse things in life”*; *[UK, 25 years, female]*

*“Try and be as positive as much as you can, don’t be miserable about it”*; *[UK, 39 years, male]*

*“Do not resign yourself to a situation…be hopeful that it will improve”*; *[NL, 54 years, female]*

*“Someone has to remain positive……I think positivity breeds positivity”. [NL, 33 years, female]*

## Discussion

This research reveals novel insights about the nature of support provided by significant others in helping those with CMP to stay at work: most studies in this field tend to document the negative or unhelpful behaviours of significant others [20, 21], or the incongruence between patient and partner ratings of pain and disability [22, 9]. In contrast, the present study demonstrates the largely positive responses of significant others, and illustrates a highly congruent ‘partnership’ approach to managing CMP in order to remain at work. The findings of this study add to our current understanding of how social support mechanisms may operate in the context of chronic pain.

The prevailing theoretical approaches explaining how significant others are unhelpful in this process are well documented. The operant model of pain [23] advances the notion that significant others have active roles in the experience of pain via reinforcement and that this explains why some pain behaviours can persist over time in the absence of underlying pathology [24]. Cognitive-behavioural models have also been developed to explain this phenomenon, whereby the perceptions and thoughts of significant others are proposed to play an important role in pain adjustment as they translate into unhelpful behaviours such as solicitousness or punishment [25]. More recent research has begun to acknowledge the importance of self-regulation in this field, which suggests that significant others’ own beliefs and meanings about pain may be particularly salient influences on their relative’s persistent pain behavior and disability [26, 27].

However, more recent theoretical explanations have attempted to describe how significant others can be helpful in this process. Here, it has been suggested that patient pain behaviours trigger intrapersonal processes in their partners to help them understand the patient’s pain behavior, and that empathic responses from significant others build intimacy, enhancing adjustment to pain [24]. This seems to align with the findings of the present study, whereby significant others described (and were described by workers as) having a positive outlook which was seen as an important factor for continued work participation. Significant others also described the positive outcomes (on both themselves and their relationship) as a result of providing support to workers. However, it should be acknowledged here that both of the study samples were self-selecting and only those couples who felt they had a strong relationship and wanted to demonstrate it may have participated in our research. Due to the relative lack of research in this field, questions still remain about this interpersonal process.

For example, does the positive and supportive nature of significant others help those with CMP to successfully self-manage and remain at work, or conversely, is it because individuals themselves have managed their CMP successfully that this produces a more positive response from significant others? Or, does the proposed increase in relationship strength as a result of empathic responses from significant others buffer the negative effects on the relationship due to one partner having CMP? Complexity can also arise when this support or empathic response, viewed by couples as an indicator of relationship strength, translates into solicitousness which has a potentially detrimental effect on pain outcomes [8, 28–30]. It has also been suggested that by providing help with everyday tasks, significant others are reducing the amount of activities for the person with pain, leaving more time for them to focus/ruminate on their condition. Furthermore, accepting this support may lead to the individual with pain to feel they are losing autonomy, thereby reducing their capacity to develop sustainable coping strategies [31], but providing (and describing) support may allow significant others to fulfill their ‘normal’ role as a caring family member in the face of an ‘abnormal’ situation [32]. Our exploratory work cannot fully examine such complex interactions, but it clearly points to areas worthy of future research.

A specific example of this complexity was observed within in the ‘connectivity’ theme, whereby significant others reported that talking about the worker’s pain was perceived to be a helpful support mechanism. Receiving emotional support is thought to be particularly helpful for those with CMP as it is said to legitimize an invisible condition [33], but difficulties can arise when significant others do not feel this is reciprocated. In this study, significant others often stated that workers did not want to talk about the pain -indeed, workers did not elaborate on this theme as much as significant others, and it could be interpreted that talking was not always helpful to workers because it focused attention on their pain. However, for significant others, talking about the worker’s pain may have also helped alleviate their own concerns, some of which were highlighted in this theme. This aligns with other research in this area and illustrates the complexities which can arise due to the different needs between those experiencing pain and those close to them: those with chronic pain are fearful of the demands their condition places on others and tend to minimize its impact, but those close to them need to have opportunities to access their own support in order to reduce the burden placed on them and help to attenuate any maladaptive appraisals of chronic pain [26, 34, 35], but also to have their feelings and experiences recognized in order to provide them with evidence they are in a strong, reciprocal relationship [36]. These findings indicate that those with chronic pain and their significant others may have different information and support needs, and that addressing both could counteract any negative outcomes related to relationship disparities.

Although it is promising that similar findings were reported in two un-related studies conducted in two different countries, it is acknowledged that the relatively small sample size means that results cannot be generalizable. Nonetheless, our sample is certainly of the size deemed sufficient for data saturation purposes in exploratory qualitative research [37, 38]. Data analysis and assimilation were subject to recommended procedures to ensure consistency and credibility, but combining un-related data from participants with different musculoskeletal pain conditions inevitably means that a certain degree of flexibility was required to reconcile features of both studies. Thus, it is recognised that findings could be vulnerable to redefinition with implications for the validity of our results, but there is no support in the literature to suggest sub-grouping by pain site is helpful when continued work participation is the aim, while there is evidence that non-specific chronic pain can be classified based on psychosocial complexity [39, 40].

Whilst the authors recognise that combining data from two studies is not without potential limitations and difficulties, we would nonetheless argue that this needs to be balanced with the need to explore and elaborate on an under-researched, yet important, topic in order to inform the design of a larger, more robust study in this area. For the secondary analysis presented here, we adhered to a number of recommendations made by other authors [14, 15]: we used similar analytic techniques as those used in the primary data set, we perceived there to be a good fit between the primary data sets and our research question and we established a productive and effective collaboration between the two original study teams. The thematic analysis approach taken lends itself well to group or team analysis as the research team collaboratively defined thematic meanings and structure in an iterative process [41]. Additionally, having been involved with one of the original two studies from which the data set was drawn meant that all authors were sensitive to the context of the work, whilst usefully able to provide a fresh perspective on other aspects of the data. It has been suggested that posing new research questions of existing qualitative data sets can generate valuable knowledge [14], and our findings do indeed usefully point to areas warranting further investigation and highlight issues of practical concern in research involving significant others. The appropriate secondary analysis of qualitative data also ensures that these richly descriptive datasets are not underused – as well as there being a strong pragmatic argument for this, we would contend that such an approach, which more fully utilises available datasets, may also be more considerate and respectful of the research participants themselves, who have generously given their time and shared their experiences.

## Conclusion

This research responds to a call for more studies to focus on the under-represented ‘social’ component of the biopsychosocial model currently applied to our understanding of CMP [42], further contributing to the emerging evidence which stresses the need to acknowledge the individual’s social context to improve clinical and work outcomes [43–45]. This will become increasingly more important due to the growing demands placed on healthcare as a result of an ageing population, as many of those with chronic health conditions will become more reliant on the support of their significant others. Recognising this however, does not diminish the challenge to clinicians and researchers in this field who often do not have the resources to tackle many of the ‘social’ influences on health and work participation. Those with CMP and a physically demanding job who do not possess the ability or qualifications to move into a less physically demanding, more flexible job will face persisting difficulties despite effective pain management or vocational rehabilitation strategies. Indeed, the participants in our studies were largely employed in professional occupations with a greater degree of job autonomy and flexibility. This in itself could have had a greater influence on their continued work participation over and above workers’ own management strategies or support from their significant others. Although the findings of this study provide useful insights into how sources of social support available to those with CMP could be capitalized upon, our work also highlights the complexity involved when incorporating the social context into the healthcare arena.

## Abbreviations

CMP: Chronic musculoskeletal pain
